# Generalized pulp canal obliteration in a patient on long-term glucocorticoids: a case report and literature review

**DOI:** 10.1186/s12903-022-02387-9

**Published:** 2022-08-15

**Authors:** Ban Jiandong, Zhang Yunxiao, Wang Zuhua, Hou Yan, Geng Shuangshuang, Li Junke, Wang Hongwei, Xu Hua

**Affiliations:** 1 Department of Stomatology, Hebei Eye Hospital, Xingtai, 054000 China; 2 Department of Haematology, The First Affiliated Hospital of Xingtai Medical College, Xingtai, 054000 China; 3 Department of Endodontics, Stomatological Hospital of Peking University, Beijing, 100010 China

**Keywords:** Glucocorticoids, Paroxysmal nocturnal haemoglobinuria, Pulp canal obliteration, Root canal therapy

## Abstract

**Background:**

The calcification of the tooth pulp is a pathological condition that occurs in response to various factors. A uncommon haematological condition known as paroxysmal nocturnal haemoglobinuria (PNH) is characterized by bouts of haemolysis, and it requires long-term use of glucocorticoids (GCs).

**Case presentation:**

A female patient who was diagnosed with PNH and had a history of long-term use of GCs came to our department for root canal therapy (RCT) for teeth 25, 26, and 27. The radiographs showed generalized pulp canal obliteration (PCO) in most of the patients. None of these teeth (25, 26, or 27) were sensitive to percussion, and they did not respond to thermal or electrical sensitivity tests. A diagnose of pulp necrosis was made for these teeth. RCT was carried out with the help of an oral microscope, and then a prosthodontic procedure was created for the teeth.

**Conclusions:**

Based on the patient’s long history use of GCs and a series of related studies, we conclude that the long-term usage of GCs contributes significantly to the onset of PCO.

## Introduction

Glide path is the key to infection control in RCT, but in clinical practice, root canals are often blocked due to pulp calcification, which makes accessing and cleaning the root canal system more difficult. Local and systemic factors may contribute to the formation of dental pulp calcification [1]. Excessive forces caused by trauma and clenching, the presence of restorations, cavity preparation, and caries are common local factors [2, 3]. Systemic factors include end-stage renal diseases [4], cardiovascular disease [5], and some long-term medications [1, 6, 7].

Ranjitkar et al. [8] believed that pulp calcification is an age-related disease. The aging process of tooth pulp leads to a decrease in fibroblast, odontoblast, and mesenchymal cells. Between the ages of 20 and 70, these cells have been reported to decrease by 50%. With age, secondary dentin accumulates gradually, pulp cavity and root canal become smaller, fat may deposit in the pulp, and calcification usually occurs at these deposits [9]. Therefore, age-related calcifications are mostly located in the medullary cavity, canal orifice, and upper canal segment. Some scholars have proposed a relationship between periodontal disease and pulp calcification [10], and pulp calcification is a common manifestation in the healing process after a traumatic injury. Dental-pulp calcification can be considered a kind of active repair. In the situation of tooth caries, periodontal disease, trauma and abrasion, and dental pulp produce a defensive reaction. In the defensive process, the formation of the calcification focus is centred on the degeneration and necrosis pulp cells. At the same time, the secretion of reactive dentin by odontoblasts is accelerated, eventually leading to the formation of pulp stones or diffuse calcification and blocking the root canal system [11, 12]. It has also been proposed that the blood clots formed after tooth injury will calcify and trigger calcification of the remaining pulp [13]. The location of calcification is mostly related to the origin of stimulation, so periodontal calcification usually begins at the root tip whereas stress stimulation calcification can occur throughout the root canal.

Other studies have shown that the application of calcium hydroxide and hydrogen peroxide can promote calcification [14, 15]. Some scholars isolated nanobacteria (CNPs) that can cause calcification of biological tissues in myeloid stones [16], and the presence of CNPs may promote the calcification of nuclear, thus forming a biological apatite structure or leading to cell-mineralization denaturation. Studies have also shown that dentine dysplasia can lead to dental pulp calcification, and the gene mutation of the bicistronic dentine sialophosphoprotein (DSPP) is one of the key variables contributing to dentine dysplasia [17].

In addition, long-term use of GCs has been correlated with calcification of the dental pulp, and a few related clinical reports have been reported [6, 18, 19]. According to Chigono et al. [19], a patient with systemic lupus erythematosus was taking oral GCs for a long time. They examined the patient’s premolar stumps histopathologically and found that nearly all samples had constricted dental pulp and found no odontoblasts.

A uncommon haematological disorder known as paroxysmal nocturnal haemoglobinuria (PNH) is characterized by bouts of haemolysis [20]. Because of its many forms and complex pathophysiology, it has piqued the attention of haematologists for more than a century [21, 22]. PNH is thought to affect 1–1.5 instances per million people globally, while this number may be greater in certain areas [23, 24]. The development of the illness into myelodysplastic syndromes, bone marrow failure, and thrombosis are further clinical signs of PNH [20]. Due to the clonal growth of a mutant haematopoietic stem cell, PNH is believed to originate from these non-erythroid characteristics (HSC) [25]. This fatal condition's biological anomaly results from a mutation in the phosphatidylinositol glycan class A (PIGA) gene, which causes a shortage of complement regulating proteins that are glycosylphosphatidylinositol (GPI)-anchored, such as CD55 and CD59, on blood cells' surfaces [23, 26].

The most common cause of death is thromboembolism, which is much more common in patients with PNH haemolysis [23, 27]. The traditional treatment for PNH [24] is symptomatic support therapy, and GCs are often administered to alleviate anaemia and perhaps reduce the frequency of haemolytic episodes. Other symptomatic supportive treatments include transfusions of red blood cells and platelets when necessary and antibiotics when infections occur.

## Case presentation

**Fig. 1:**
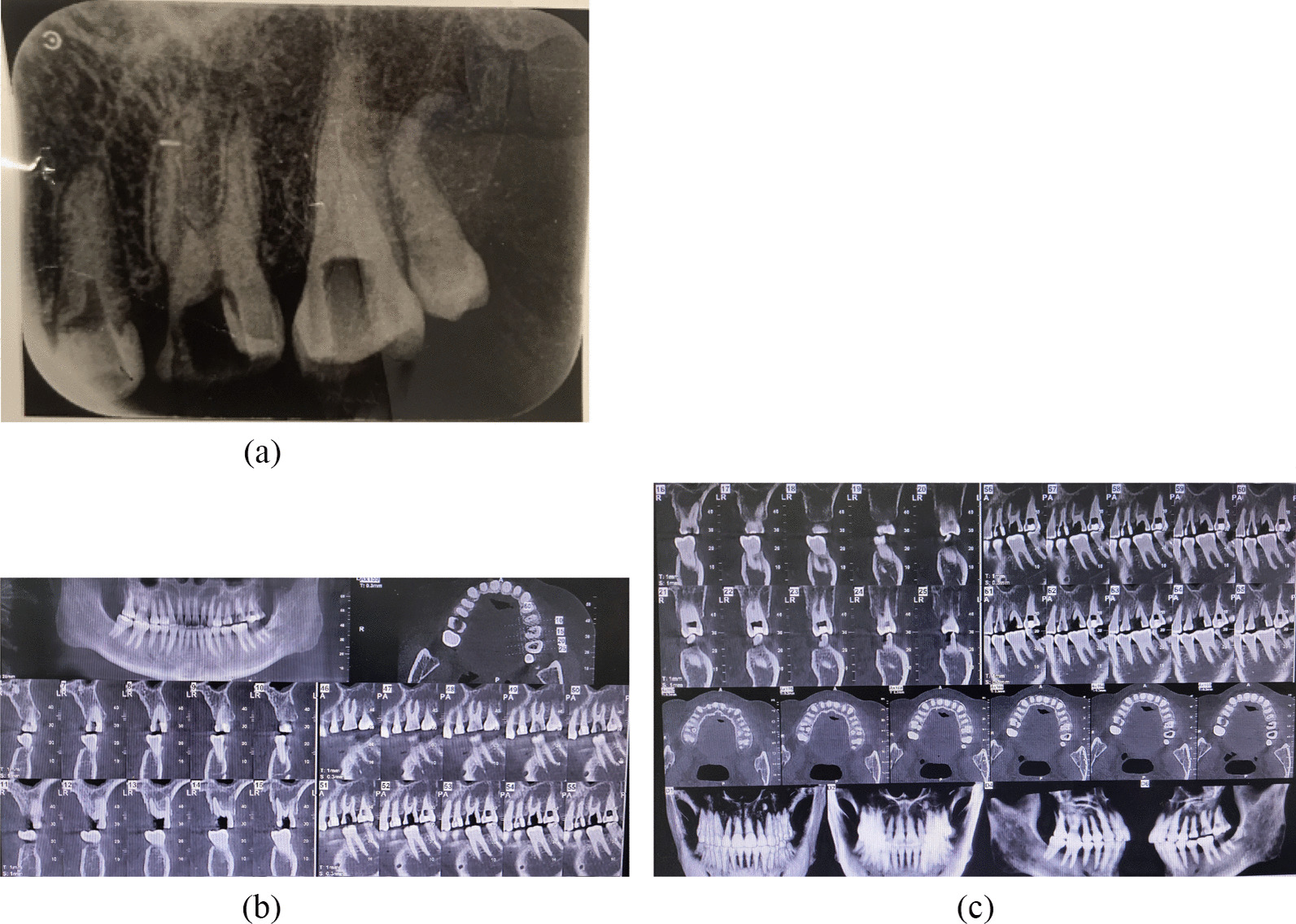
Preoperative dental and CBCT radiographs show generalized pulpal obliteration. The pulp chamber is completely absent in almost all maxillary and mandibular teeth

**Fig. 2:**
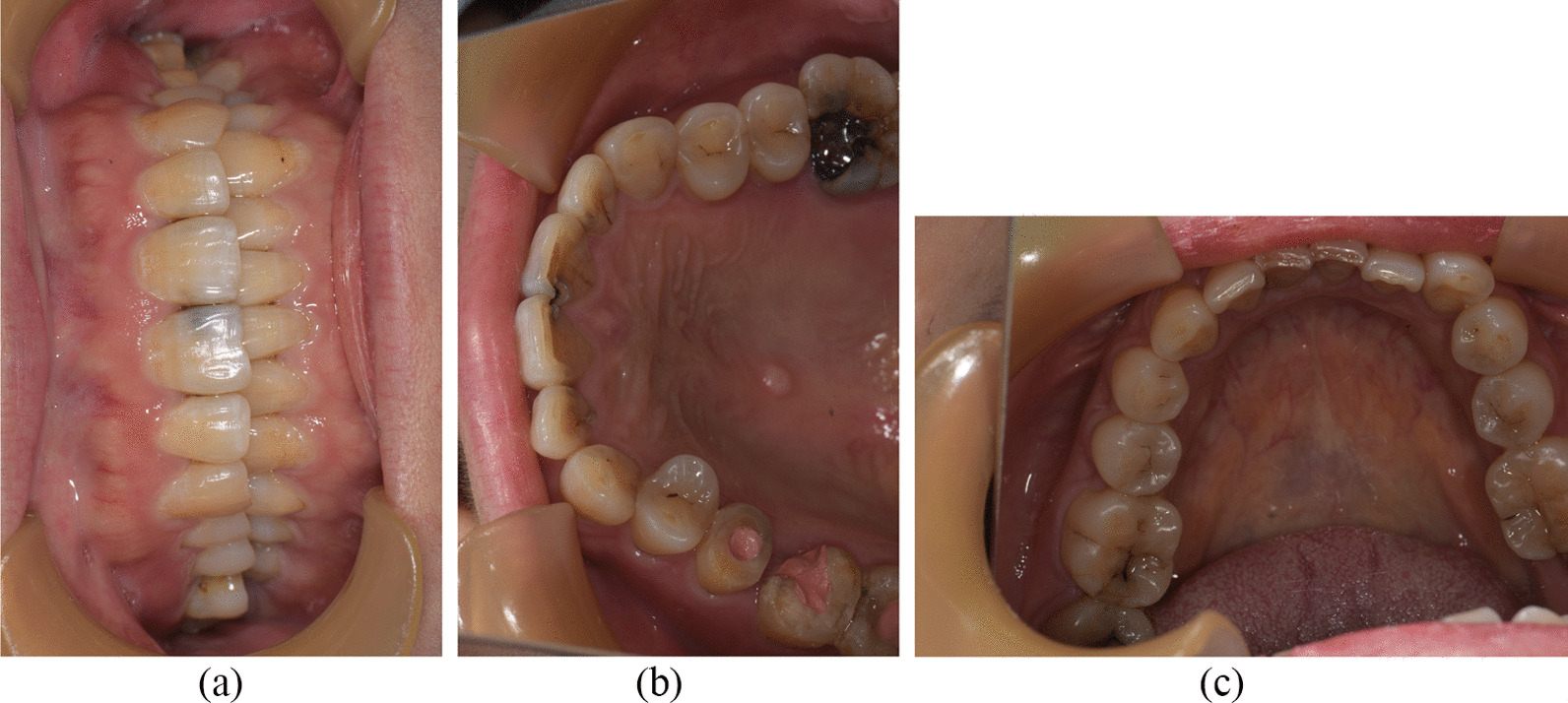
Occlusal photograph and occlusal surface images of the upper and lower teeth. The teeth’s shape and colour are normal

**Fig. 3:**
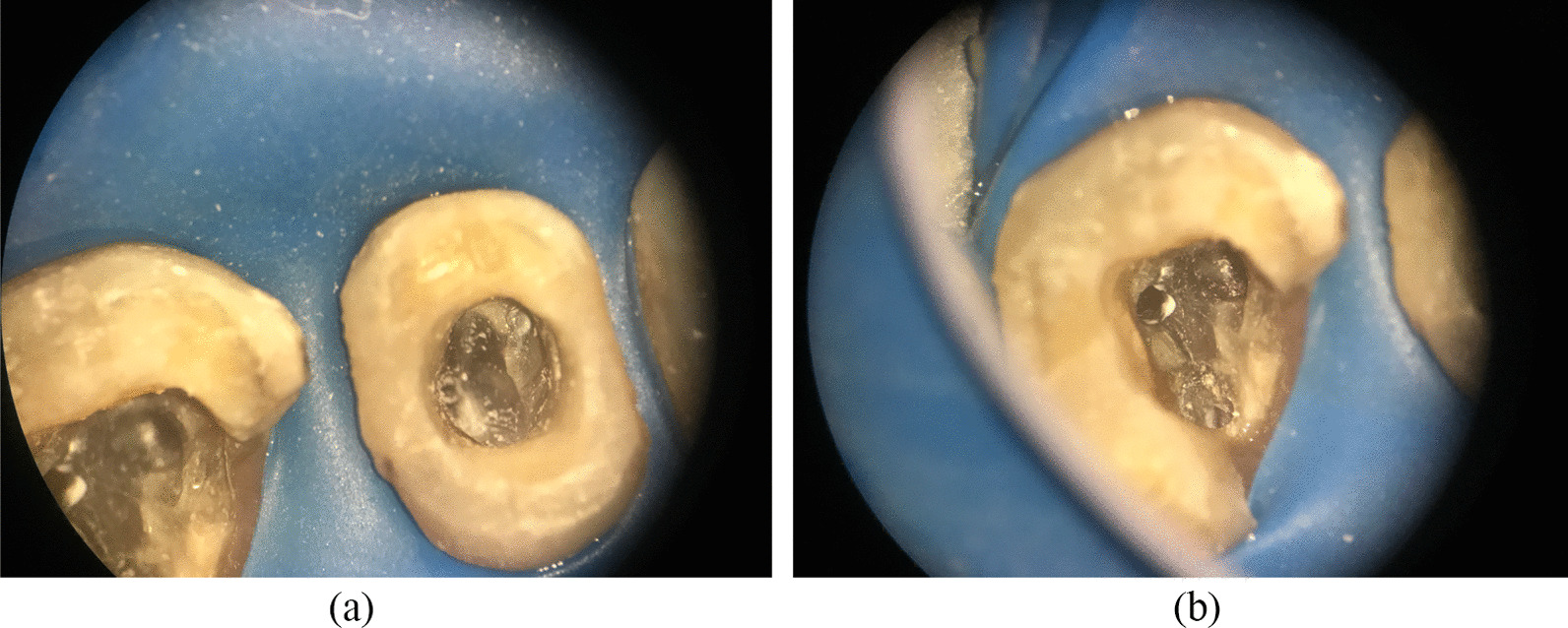
Pulp canals have narrowed or disappeared

**Fig. 4:**
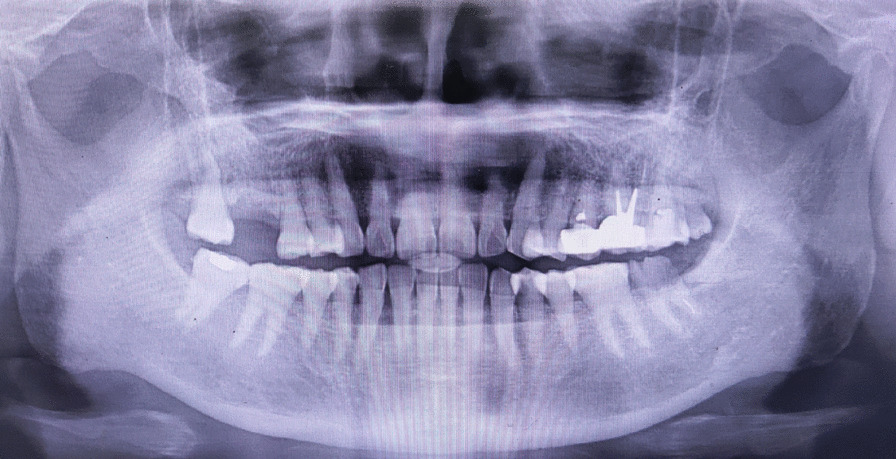
Panoramic radiograph taken in December 2020 showing a steady state of the treated teeth and the generalized pulpal obliteration of all teeth

**Fig. 5:**
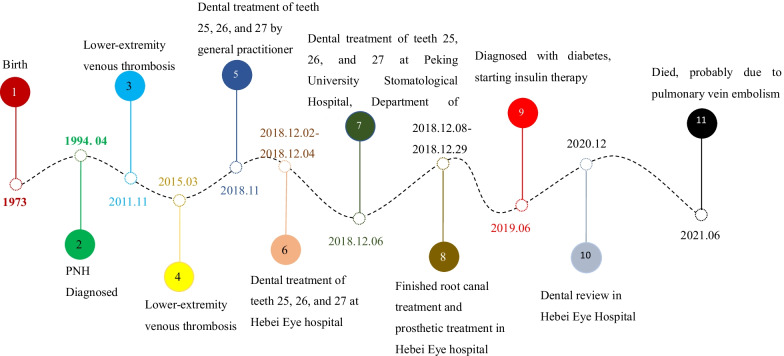
The entire course of the patient’s diseases

The female patient was born in 1973 and was diagnosed with PNH at the age of 21 in 1994. Since then, she has taken GCs (methylprednisolone, about 24-50 mg/day) for more than 20 years, mostly without interruption although with varying doses. The patient also took ciclosporin for a short time when she was diagnosed with PNH, and she took aspirin and calcium tablets intermittently. In 2011 and 2015, the patient had been hospitalized twice for lower-extremity venous thrombosis. In 2018, the patient experienced spontaneous and nocturnal pain in the upper left posterior teeth, and she visited a general practitioner. Although the toothache symptoms were relieved after pulp access, the general practitioner was unable to access or debride the root canals, and the patient was advised to visit our department. Teeth 25, 26, and 27 had been treated. None of these symptomatic teeth were sensitive to percussion, and testing of their thermal or electrical sensitivity revealed that they did not react. A periapical radiograph showed no obvious root canal image of the symptomatic teeth (Fig. 1a). A diagnosis of pulp necrosis was made for these teeth. The root canals of the symptomatic teeth were examined using cone-beam computed tomography (CBCT) to see whether they were entirely or partly obstructed. The CBCT images revealed that the symptomatic teeth were completely blocked and generalized PCO of the patient’s teeth had occurred (Fig. 1b, c). The patient was in a normal occlusion relationship, with no sign of wear on any of the teeth’s occlusal surfaces (Fig. 2). The patient had no history of trauma or orthodontic or surgical interventions in her teeth or jaws. The symptomatic teeth’s root canals were so obstructed that they could not be accessed or debrided. Then the patient was transferred to another endodontics expert. However, even with the help of an oral surgery microscope and oral ultrasonic equipment, only tooth 26’s mesial buccal and distal buccal canals were accessed and debrided (Fig. 3). Calcium hydroxide was applied to the symptomatic teeth for two weeks, and the AH-plus sealer and gutta-percha were used to obturate the debrided canals, and the unlocated canals went without any further treatment. Then teeth 25 and 27 were filled with composite resin and a crown was made for 25. As for tooth 26, a post–core crown was made. The patient attended a followed-up visit two years later and showed no discomfort or periapical inflammation of the treated teeth (Fig. 4). In 2021, the patient died unexpectedly at the age of 48. The probable cause of death was a pulmonary embolism due to a microthrombus, according to her haematology doctor. Figure 5 shows the entire course of the patient’s diseases.

## Discussion and conclusions

GCs are widely used to treat arthritis, chronic asthma, allergies, and autoimmune diseases and following organ transplants [28]. However, long-term use of GCs may cause an increased risk of infection, osteoporosis, retardation of growth in children, and disturbances in wound healing [28, 29]. The incidence of GCs’ adverse reactions depends on the dose and, to a greater degree, the duration of treatment [28]. One possible side effect of GC therapy in the dental field is extensive narrowing of dental pulp [19].

In 1966, dental-pulp calcification was seen in the adult cortisone-pretreated rats' incisors, according to Anneroth et al. [30]. The odontoblasts' differentiation was reduced and there was a clear disarray in the cortisone-treated rats. Rats receiving treatment had more undifferentiated odontoblasts than untreated rats. The pulp tissue looked to be exceedingly cell-rich overall in the cortisone-treated rats, and the number and breadth of blood vessels increased. In certain areas of the pulp, the researchers saw an excessive growth of a hard tissue that resembled bone and was full of cellular and vascular inclusions. The pre-dentinal zone, which was substantially expanded and had an uneven border facing the dentin, also included comparable inclusions, which they also noted. In another animal experiment, Näsström et al. [4] observed that in the molars of adult GC-pretreated rats, new dentin was forming, but there was no dentin development observable in the control group. However, the results of animal experiments were not unanimous concerning GCs’ effect on dentin formation. Ball et al. [31–33] observed in three trials that rats given subcutaneous injections of corticosteroids had narrower dentin walls, resulting in larger pulp chambers in the incisors. After administering corticosteroids to freshly weaned rats, Johannessen et al. [34] discovered that dentin development in the molars was suppressed. However, primary dentin development seemed to be finished somewhere between the 12th and 35th day of life [35–37]. There seems to be a lot of diversity in the daily dentin apposition in the molars of newborn rats. Secondary dentin production begins on day 35 of the rat's life and is seen in the crown pulp under the cusps [36, 38] when secondary dentin is stimulated by attrition. Comparing the age of experimental rats in the two studies [4, 34], the difference in results may occur because Young rats (21 days) with primary dentin development underway were employed by Johannessen [34], while mature rats were used by Näsström (3.5–5 months). Moreover, it is possible that the disparate effects seen in investigations on the impact of corticosteroids on rat incisors and molars [31–34] and molars [4, 34] were brought on by the use of various corticosteroid dosages. As opposed to this, according to the results of the energy-dispersive X-ray microanalysis [4], the dentin caused by GCs was found to have the same calcium and phosphorus composition as normal dentin in the rat molars of the control group. This finding may indicate that treatment with GCs did not affect the mineralization process, and as a result, the quality of the GC-induced dentin may be equal to that of normal dentin in calcium and phosphor. A scanning microscopy study also revealed that the morphology of the GC-induced dentin in rat incisors was similar to that of naturally occurring dentin [4]. Näsström et al. [4] inferred that GCs might exceed protein synthesis and would therefore induce accelerated production of the matrix in the predentin zone. The mineralization rate could be normal, as seen in the experimental studies on rat incisors, but as the corticosteroids received continuously activate the odontoblasts, the mineralization process will proceed even in mature, formerly resting odontoblasts, as was noted in the experimental study on rat molars.

In normal adults, as the tooth ages, the coronal and root regions of the pulp chamber gradually continue to experience dentin mineralization [39]. In 51 individuals with renal disorders, the radiographic narrowing of the tooth pulp chamber was examined [40]. The findings showed that there appeared to be a relationship between dental pulp chamber narrowing, the quantity of corticosteroids received, and the pharmacokinetics (total plasma prednisolone clearance) of these drugs. This was done since there was no way to link a constriction of the tooth pulp chamber to a particular renal illness. On the other hand, an individual response to the corticosteroid treatment probably occurs [4, 40]. In a few patients, a relatively low total dose of corticosteroids started a productive reaction in the odontoblasts and caused narrowing of the dental pulp chamber whereas in others, a high total dose caused no dentin formation. The explanation for the different reactions might be the renal failure and its influence on hormone metabolism and therefore on dentin formation, which is a complex series of events not completely investigated.

Shinozuka et al. [41] compared the narrowing of dental-pulp cavity between patients under long-term steroid treatment (the steroid group) and patients who were not receiving steroids (the non-steroid group). The results indicated that the long-term administration of steroids was the main reason for the greater narrowing of dental pulp cavity in the steroid group. In our case, the patient had taken GCs for more than 20 years, and we think that the long-term administration of GCs was likely a major factor causing generalized PCO.

On the other hand, as described in this case, teeth with PCO come into the high-difficulty category of the American Association of Endodontists Case Assessment criteria if RCT is necessary [42]. In clinical practice, PCO is usually caused by dental trauma, and it usually affects young adults’ anterior teeth [43, 44]. A serious complication in teeth with PCO is pulp necrosis. Following an observational period of between 3.4 and 16 years on average, the incidence of the problem in permanent teeth with PCO varied from 1 to 16% [45–48]. The clinical crown of these teeth may also get discolored, becoming darker than the surrounding teeth in certain cases. The increasing dentine thickness, which causes the crown's translucency to diminish, is the cause of this cosmetic issue [46, 49].

Vinagre et al. [48] developed an improved clinical decision-making methodology for the treatment of PCO teeth based on the findings of a systematic review and the most current literature. This clinical decision-making algorithm demonstrated that therapy recommendations are based on clinical and radiographic symptoms and indicators, including discolouration signals.

The most common clinical strategy used for PCO teeth was watchful waiting. Additionally, the prophylactic RCT technique should not be employed as a preventative measure or as a first line of treatment for discolored, asymptomatic PCO teeth, according to the research [48]. In these situations, external bleaching needs to be the first approach used to remedy cosmetic issues. When there were indicators of periapical disease on radiographs or in symptoms, an endodontic approach was advised. Send them to an endodontist if required. Clinicians may choose for guided access or, if it's feasible, a traditional approach depending on the circumstances [50, 51]. Based on the results, endodontic microsurgery [52] or even an intentional re-implant when the surgery is not feasible [53, 54] is advised in the event of failure. The final resort is tooth extraction, which must be followed by an appropriate rehabilitation therapy.

### Conclusion

Although the effects of PNH and other medications cannot be ruled out, based on the patient’s long history of GC use and a series of related studies, we conclude that the long-term usage of GCs contributes significantly to the onset of PCO. In clinical work on patients receiving GCs special care, a clinical decision-making algorithm must be used in therapy planning.

## Data Availability

The corresponding author may provide the datasets used and/or analyzed during the present investigation upon reasonable request.

## Abbreviations

GCs: Glucocorticoids.
CNPs: Nanobacteria
NTs: Neurotrophins
PNH: Paroxysmal nocturnal haemoglobinuria
HSC: Haematopoietic stem cell
GPI: Glycosylphosphatidylinositol
PIGA: Phosphatidylinositol glycan anchor biosynthesis class A gene
CBCT: Cone-beam computed tomography
